# Current Status of Gout Arthritis: Current Approaches to Gout Arthritis Treatment: Nanoparticles Delivery Systems Approach

**DOI:** 10.3390/pharmaceutics17010102

**Published:** 2025-01-14

**Authors:** Yedi Herdiana, Yoga Windhu Wardhana, Insan Sunan Kurniawansyah, Dolih Gozali, Nasrul Wathoni, Ferry Ferdiansyah Sofian

**Affiliations:** 1 Department of Pharmaceutics and Pharmaceutical Technology, Faculty of Pharmacy, Padjadjaran University, Sumedang 45363, Indonesia; y.w.wardhana@unpad.ac.id (Y.W.W.); insan.sunan.kurniawansyah@unpad.ac.id (I.S.K.); dolih.gozali@unpad.ac.id (D.G.); nasrul@unpad.ac.id (N.W.); 2 Department of Pharmaceutical Biology, Faculty of Pharmacy, Padjadjaran University, Sumedang 45363, Indonesia; ferry.ferdiansyah@unpad.ac.id

**Keywords:** preventive approach, nanoparticle delivery systems, lifestyle modification, uric acid, quality of life

## Abstract

The deposition of monosodium urate (MSU) crystals within joint spaces produces a painful inflammatory condition known as gout, a specific form of arthritis. The condition calls for a combined curative and preventive management model. A new development in the approach to gout is that of NLRP3-targeted biologic agents, such as monoclonal therapies, to provide more accurate treatment by blocking specific pro-inflammatory cytokines. Nanoparticle drug delivery enhances biological availability and delivery to targets, which may increase therapeutic efficacy and decrease general toxicity. The preventive approach again cannot be ignored, mainly keeping up certain modifications in diet and weight, along with pharmacological therapies to reduce uric acid (UA) levels and to decrease the frequency of acute attacks. The advancement of genetic profiling of patients and biomarker discoveries drives the trend towards building individualized medicine and care, quickly gaining ground as the most effective method of delivering treatments to individual patients, moving away from one-size-fits-all treatments. The following paper aims to provide an updated account of the management of gout with a focus on recent developments, in order to enhance these approaches, the quality of life for patients with gout, and the standard of gout treatment.

## 1. Introduction

Among inflammatory arthritic conditions, Gout Arthritis (GA) ranks as a prevalent disorder, impacting between 0.02% and 6.80% of adults globally. Gout is a clinical manifestation of hyperuricemia, with Serum Uric Acid (SUA) greater than 6.8 mg/dL. This condition results when too much UA is produced, or its elimination is reduced; monosodium urate (MSU) deposits in joints and soft tissues to cause acute and chronic inflammation [1,2,3,4,5,6,7]. The significant factors contributing to the development of gout include genetic predisposition, lifestyle choices, metabolic factors, and comorbid conditions. Genetic factors are essential, as specific genetic variants can affect the body’s ability to metabolize UA [3,4,5,8].

Lifestyle factors, such as excessive consumption of purine-rich foods (e.g., red meat, seafood), alcohol, organ meats, glutamate-rich foods, RNA-rich foods, and sugary beverages, increase UA production and decrease excretion [1,9]. Obesity and metabolic syndrome worsen the risk of developing gout by increasing UA production and reducing its renal clearance. High UA levels (hyperuricemia) become increasingly common as kidney function declines, affecting 11% of people with normal kidney function but rising to 80% in those with stage 4 chronic kidney disease (CKD). Conversely, poor kidney function (CKD stage 2 or higher) is found in 70% of people with gout and 50% of those with hyperuricemia, creating a cycle in which each condition can worsen the other. This bidirectional relationship occurs because damaged kidneys cannot effectively remove UA from the body, leading to its accumulation [10,11,12]. UA is characterized by painful, intermittent, and often recurrent episodes of arthritis due to fluctuations in UA levels during different phases of the disease [1].

MSU crystals initiate an inflammatory process mediated by innate immune mechanisms, leading to both engagement of neutrophil-to-lymphocyte ratio family pyrin domain, containing 3 (NLRP3) inflammasome, and the production of inflammatory cytokines, notably IL-1b [1,2,5]. Therefore, recent studies have highlighted NLRP3 inflammasome as a potential therapeutic target due to its role in triggering inflammation and tissue damage. The NLRP3 inflammasome has also been proposed as a promising diagnostic biomarker and therapeutic target for developing new drugs and early diagnosis of UA [5,13,14]. Cell death mechanisms, such as pyroptosis, neutrophil extracellular trap formation (NETosis), necroptosis, and apoptosis, are also involved in this reaction [3].

Acute gout flares are typically treated with anti-inflammatory medications, such as Nonsteroidal Anti-inflammatory Drugs (NSAIDs). At the same time, long-term management often relies on urate-lowering therapies, particularly xanthine oxidase inhibitors (XOIs). XOIs are generally considered adequate, safe, and well-tolerated by most patients in the long-term management of uric acid levels [15,16,17]. However, existing therapies typically provide only symptomatic relief and can cause side effects [3,18]. Effective management of gout faces several challenges, including the need for rapid and effective treatment during acute gout attacks, where traditional medications may not provide sufficient relief and may be accompanied by significant side effects.

The administration of urate-lowering agents, specifically allopurinol, febuxostat, probenecid, sulfinpyrazone, and benzbromarone, can increase the renal metabolic load, raise the risk of nephrolithiasis, enhance uric acid secretion, and reduce purine metabolism. Treatment efficacy is compromised when patients fail to maintain consistent long-term medication compliance. While inflammatory conditions can be managed with colchicine, corticosteroids, and nonsteroidal anti-inflammatory medications, these agents present adverse effects. The pharmaceutical development process remains gradual, and current therapeutic options inadequately address all gout phases. Various constraints restrict the broad application of these medications [19]. Long-term management requires control of UA levels to prevent future attacks, which can be difficult due to issues with adherence to treatment, dietary changes, and the need for ongoing monitoring.

Advances in nanotechnology offer promising new approaches to the improvement of gout management. The emergence of nanotechnology has created novel opportunities for enhancing drug formulations’ pharmacological properties and clinical effectiveness. Various nanostructured delivery systems—including polymeric nanoparticles, liposomes, SLNPs, and nanoemulsions—have remarkably improved when incorporating traditional herbal medicines, offering enhanced characteristics and precise targeting capabilities [20]. Nanoparticles, which are 1 to 100 nanometers in size, have significant potential to improve drug delivery systems, reduce inflammation, and modify the behavior of urate crystals. They allow targeted therapeutics delivery directly to the affected joint, increasing treatment efficacy and minimizing systemic side effects [21]. Current treatments, including anti-inflammatory and uric acid-lowering drugs, often face challenges, such as poor solubility, rapid clearance, and limited bioavailability, which can reduce their therapeutic efficacy [22,23]. Nanoparticles offer a promising solution by enhancing drug bioavailability through improved solubility, protection from premature degradation, and targeted delivery to inflamed joints [24,25,26]. This can lower required dosages, minimize systemic side effects, and enable sustained therapeutic outcomes, reducing administration frequency and improving patient compliance [27,28]. Emerging therapies, such as IL-1 inhibitors (canakinumab, anakinra, rilonacept), offer new options for managing gout attacks, although some are not yet FDA-approved. Current treatments for gout have limitations and challenges that must be overcome, especially given the chronic nature of the disease, which often requires a multifaceted approach.

This manuscript aims to provide an in-depth exploration of the role of nanoparticles in gout treatment, detailing their mechanisms of action, potential benefits, and prospects. By synthesizing recent developments and identifying gaps in the current knowledge, this work offers valuable insights into how nanotechnology can revolutionize the management of gout, ultimately contributing to more effective, targeted, and personalized treatment options for patients.

## 2. Overview of Gout

### 2.1. Pathophysiology

The purine metabolic derivative, UA, demonstrates diverse biological roles spanning physiological and pathological conditions, including immune response modulation, blood pressure maintenance, neural protection, antioxidant activity, nitric oxide modulating, and anti-aging [15,29,30]. However, excessive UA levels, whether due to overproduction or impaired renal and intestinal excretion, lead to hyperuricemia, the primary cause of gout [3,8,12,18]. The dynamic balance of urate in circulation reflects the intricate coordination between its synthesis and removal, facilitated by specialized transport mechanisms within different epithelial tissues and cells [31]. Elevated UA levels form MSU crystals, accumulating in joints and triggering GA [2,6,9,32]. Hyperuricemia, a significant condition leading to gout, typically develops from under-excretion of urate (excretion < 330 mg/d), while urate overproduction (excretion > 600 mg/d) accounts for fewer cases. Acute gout flares may be initiated by specific stimuli—notably excessive eating, strenuous physical exertion, cold temperature exposure, and alcoholism—which promote heightened ATP degradation to AMP, consequently increasing UA production [6,18]. UA solubility varies with urinary conditions (concentration, volume, pH) and excretion rates. Below pH 5.5, undissociated UA precipitates form crystals. Risk factors for UA stones include acidic urine, reduced volume, elevated UA levels, gout predisposition, and high purine intake [33].

**Figure 1:**
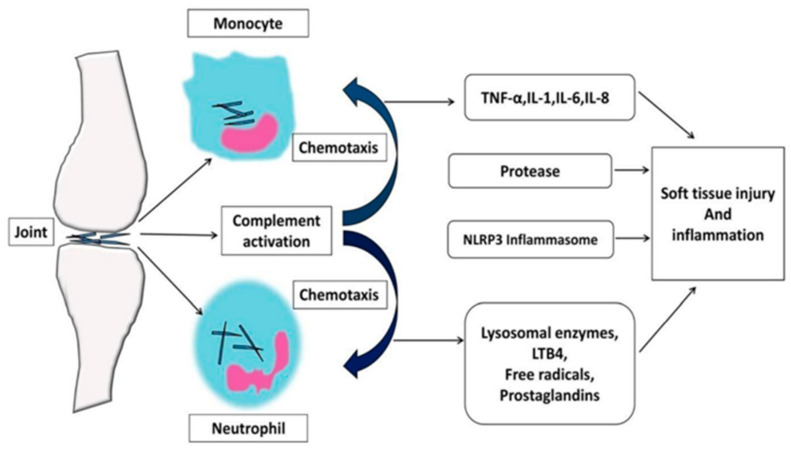
Pathophysiology of GA inflammation. (IL-1: Interleukin 1, IL-6: Interleukin 6, IL-8: Interleukin 8, LTB4: Leukotriene B4, NLRP3: Nod-like receptor protein 3, TNF-α: tumor necrosis factor-alpha) [9].

The development of gout follows four distinct pathophysiological stages: (1) hyperuricemia; (2) formation and deposition of sodium urate crystals; (3) acute gout flares characterized by inflammatory reactions; and (4) chronic gout and tophi formation, which can lead to irreversible bone erosion and joint deformity [25]. The formation of MSU crystals from UA in gouty conditions generates pain through multiple inflammatory mechanisms, including complement system engagement, immune cell activation, and the induction of cell death via necrosis [6,34]. The pathophysiological events in gout involve the phagocytosis of urate crystals by synoviocytes, which then secrete inflammatory mediators, such as interleukin-1 (IL-1), leukotriene B4 (LTB4), prostaglandins, and tumor necrosis factor-alpha (TNF-α). The effects of urate extend to multiple immune system components, encompassing alterations in cytokine profiles, disrupted chemotactic processes, modified cellular differentiation, and activation of immune cells via intrinsic cellular stimulation, leading to the conditions previously outlined [35]. These mediators stimulate polymorphonuclear leukocytes (PMN) and mononuclear phagocytes (MNP), leading to an intense inflammatory response. MSU crystals are pro-inflammatory and can initiate, amplify, and sustain this response, with IL-1β playing a significant role in the inflammatory activity associated with crystal deposition in gout patients. Gout flares initiate when MSU crystals trigger NLRP3 inflammasome activation, leading to IL-1β secretion, whereas the condition’s resolution phase relies heavily on clustered neutrophil extracellular trap structures [36]. The development of GA involves NET-mediated release of inflammatory mediators that amplify the inflammatory response. In contrast, NET aggregation functions to contain and degrade both inflammatory cytokines and MSU crystalline structures [6]. The disease process represents a disrupted metabolism and convergence of inflammatory responses, as shown in Figure 1 [37].

Kidney function is crucial in hyperuricemia, as the kidneys are responsible for UA excretion. Impaired kidney function, whether due to CKD or hereditary factors, can lead to UA accumulation [38]. Interventions to lower UA levels show promise in decelerating CKD progression and reducing urinary protein and albumin excretion [39].

Genetic abnormalities affecting purine metabolism enzymes can also increase UA production, while hyperuricemia may develop without a clear underlying cause, indicating potential unidentified factors [40,41]. Enzymatic systems governing purine metabolism operate under strict regulation to ensure balanced synthesis and degradation at the cellular level. A key distinction exists between humans, who produce UA as their end product, and most mammals, which express uricase to transform UA into the more easily eliminated allantoin [42].

When insufficiently treated, gout can transform from episodic inflammatory events into chronic arthritis, characterized by joint deterioration, structural abnormalities, and the emergence of tophaceous formations—subcutaneous accumulations of crystallized urate [43,44,45]. Chronic gout can result in persistent inflammation and joint destruction, increasing the risk of comorbid conditions, like kidney stones and cardiovascular diseases.

Current gout therapies face significant limitations, primarily due to challenges such as poor drug solubility, rapid systemic clearance, and non-specific distribution, which result in suboptimal drug concentrations at the target site. These issues often necessitate higher doses, increasing the risk of side effects and reducing overall therapeutic efficacy [22,23]. Nanoparticle-based drug delivery systems offer a promising solution by improving the solubility of poorly water-soluble drugs, protecting them from premature degradation, and facilitating more precise targeting to the inflamed joints. These systems can potentially enhance therapeutic outcomes by prolonging drug retention at the site of action while minimizing systemic exposure, thus reducing the likelihood of adverse effects and improving the safety profile of gout treatments [46,47,48].

### 2.2. Management of Gout

#### 2.2.1. Diagnostics of Uric Acid Disorders

Diagnosis combines clinical assessment and laboratory tests, with joint aspiration and synovial fluid analysis being critical. The diagnostic confirmation of gout occurs through polarized light microscopic observation of MSU crystals, which appear as needle-like structures with negative birefringent properties. Recent diagnostic advances include dual-energy computed tomography (DECT) and ultrasound, which can detect MSU crystal deposits and provide visual evidence of gout-related inflammation [49,50].

#### 2.2.2. Pharmacological Management

The present treatment guidelines provide recommendations for the appropriate treatment of acute gout, management of the inflammasome pathway during the inter-critical period, and prevention of chronic complications [51].

1. Curative Treatment

Gout foists a significant burden on our healthcare system and the patients it affects. Although we have many oral urate-lowering medications to treat these patients, a considerable degree of treatment failure and refractory disease persists [18]. Therapeutic approaches to lowering UA levels in patients with gout involve various medications, including xanthine oxidase (XO) inhibitors such as allopurinol and febuxostat, as well as uricosuric agents, like probenecid and benzbromarone [20]. Xanthine oxidoreductase inhibitors belong to two classes: (I) purine analogs, such as allopurinol, oxypurinol, or tisopurine, and (II) others, such as febuxostat and topiroxostat [52]. Natural XO inhibitors are also being explored as safer alternatives; in vitro and in silico results divulge that phenolic compounds have a strong potential to lower UA levels via interacting with the XO enzyme and can be used to combat hyperuricemia [53]. Therapy selection should be personalized based on clinical conditions and potential side effects. For example, uricosuric agents, while effective, may pose a risk of kidney stones and require careful monitoring. Long-term use of allopurinol can lead to severe hypersensitivity, especially in individuals with the HLA-B*5801 allele. Conversely, benzbromarone has limitations in clinical efficacy due to side effect risks, whereas the long-term safety of febuxostat remains a topic of discussion. Febuxostat is indicated for patients with hyperuricemia and gout, particularly for those who experience allergic reactions to allopurinol. For patients with severe renal impairment, the American College of Rheumatology (ACR) recommends a low dose of febuxostat (40 mg/day) [15].

For managing acute gout flare, the ACR guidelines categorize the treatment into three options: colchicine, non-selective NSAIDs, and glucocorticoids. Despite its long-term use, colchicine’s close safety margin raises concerns about serious adverse effects. Chronic use of colchicine at a low dose with NSAIDs may increase efficacy and decrease adverse effects [8]. Glucocorticoids are also appropriate for patients who cannot take oral drugs [6].

The traditional treatments for gout are NSAIDS, colchicine, and corticosteroids, which act through the inhibition of different inflammation pathways. NSAIDs decrease the synthesis of COX and prostaglandin, and colchicine, on the other hand, has an action on neutrophils by blocking tubulin polymerization, thus reducing the appearance of neutrophils at the site of inflammation. Colchicine also exhibits an anti-inflammatory effect by preventing the action of adhesion molecules, such as E-selectin, on neutrophils, thus preventing chemotaxis. Despite being relatively safe when used for acute attack management, it is quite toxic and, therefore, ranks second to NSAIDs [54,55,56].

Corticosteroids also have broader immunomodulating properties because they suppress the activities of inflammatory transcription factors, including NF-kB and AP-1 [18]. Prednisone and methylprednisolone are corticosteroids commonly prescribed for short-term treatment of inflammation and potentially severe symptoms or signs; however, they are associated with the development of many long-term adverse effects, including hypertension, osteoporosis, and diabetes [57,58,59].

Gout is a connective tissue disease brought about by crystal-forming MSU through hyperuricemia, leading to inflammation through the NLRP3 inflammasome pathway in immune defense cells. The activation of the NLRP3 inflammasome in the inflammatory process mediated by MSU crystals has introduced new treatment options for gout [13]. This inflammasome is essential for pyroptosis, a type of apoptosis resulting from inflammation. In addition, other types of cell death, including NETosis, are implicated in gout; neutrophils form extracellular traps (NETs) to ensnare MSU crystals [3]. Pyroptosis through the NLRP3 inflammasome kills inflammatory cells and secretes IL-1β, beginning a stream of inflammatory molecules, whereas NETosis, a neutrophil process, opposes morbid inflammation by forming extracellular DNA structures called NETs. Other types, including apoptosis and autophagy, have also been discussed concerning gout, but more investigations are required to reveal their connexions. Potential closing gaps should be looked at in the future: effects of different types of cell death and their interconnection with gout pathogenesis [3]. This inflammasome subsequently induces interleukin-1 beta (IL-1β) release, playing a crucial role in the acute inflammatory response.

On the other hand, biological therapies, such as IL-1 antagonists, are increasingly being developed. Anakinra, an IL-1β receptor antagonist, demonstrates rapid and complete pain relief without side effects in patients who fail conventional therapy. Randomized controlled trials also show Anakinra’s efficacy in treating acute gout attacks. Rilonacept, a soluble receptor decoy, binds to both IL-1α and IL-1β to block their interaction with the native receptor, preventing cell surface receptor activation. Rilonacept is a recombinant human protein consisting of the human IL-1 receptor and IgG1 Fc, with a half-life of approximately one week. Clinical trials indicate that Rilonacept is significantly effective in preventing acute gout flares among patients initiating urate-lowering therapy with allopurinol. However, another study showed that adding Rilonacept to indomethacin treatment or using Rilonacept alone did not provide significant additional pain relief compared to indomethacin alone in managing acute GA [1,13].

Canakinumab, an IL-1β-specific antibody, surpasses conventional treatments in acute gout management, offering extended dosing intervals through a prolonged half-life. Its selective IL-1β inhibition reduces flare recurrence, providing therapeutic alternatives for patients with contraindications to standard medications [2,18].

Gut microbiota also play a significant role in UA metabolism. Dysbiosis due to a microbial imbalance in the gut can increase UA production and exacerbate gout symptoms. Managing intestinal microbial composition via various approaches, including diet modification, prebiotic and probiotic supplementation, and fecal microbiota transplantation (FMT) procedures, demonstrates potential benefits in lowering UA concentration and alleviating gout manifestations [6,31].

However, for patients who have large-footer tophi or joint deformities, alternatives to pharmacologic therapies include minimally invasive arthroscopic surgery. This procedure appears to have enabled the maneuver of turning down intra-articular pressure, besides removing unwanted tissue, with fewer side effects than conventional surgery [6,44].

2. Preventive Treatment

It is also relevant to monitor the levels of UA periodically to diagnose the effectiveness of therapy and guarantee that a level of UA does not exceed permissible limits. Such monitoring enables dose adjustments before complications or future gout attacks are experienced. Due to the systemic nature of this disease and UA level variability in the four different phases of this disease, people experience frequent or recurrent attacks [8].

Therefore, the first treatment objectives are to prevent acute gout attacks related to serum urea and manage the target levels of UA that cause recurrent episodes. For allopurinol, the dose range is between 100 and 800 mg/day, or febuxostat can be used, with a dose range of 40–80 mg/day and, occasionally, in conjunction with a uricosuric agent to achieve a target UA level. Dose adjustments using the allopurinol preparation are essential, especially in patients with these renal instabilities, to avoid potential side effects.

Some attacks are referred to as repeated or recurrent attacks, and it is known that GA has existed through several phases of unstable UA levels. The pharmacological management of gout aims to control acute attacks and lower UA levels [8]. Long-term therapy involves the use of urate-lowering medications, such as allopurinol, febuxostat, and uricosuric agents (e.g., probenecid). Allopurinol functions by inhibiting the enzyme xanthine oxidase, thereby reducing UA production, and preventing the formation of MSU crystals. Allopurinol is effective and widely used as a first-line therapy, with its main advantages being long-term efficacy and affordability. However, it may cause allergic reactions, such as rashes and, in rare cases, Stevens–Johnson syndrome. Allopurinol also has potentially serious side effects in patients with renal disease, necessitating dose adjustments in patients with impaired kidney function.

The advancement of treatment options necessitates better educational strategies for patients and healthcare providers regarding allopurinol usage, mainly to prevent AHS complications, which can substantially diminish the life quality of gout sufferers undergoing this treatment [18].

As a more recent treatment option, febuxostat serves as a substitute for allopurinol-sensitive individuals. This medication functions similarly by blocking xanthine oxidase activity and successfully reduces UA concentrations. Nevertheless, research indicates potential cardiovascular complications, limiting its use in patients with cardiac conditions [60]. As an innovative selective XOi with a non-purine structure, febuxostat undergoes primary hepatic metabolism with dual excretion pathways—renal and fecal—thus reducing kidney stress. However, its safety profile in severe CKD remains controversial, while optimal treatment protocols for dialysis patients, including the timing of urate-reduction therapy, lack clarity. Hepatic dysfunction is the most common side effect, requiring regular monitoring of liver function. Given its recent introduction to clinical practice, febuxostat administration demands cautious implementation and vigilant adverse reaction surveillance [18]. In chronic UA management, XO inhibitors, specifically allopurinol and febuxostat, demonstrate superior therapeutic value compared to uricosuric agents, such as probenecid [60]. Current research indicates that these medications may provide additional benefits for cardiovascular wellness, kidney performance, and blood sugar regulation, while remaining economically accessible [16].

The uricosuric drug class, comprising probenecid, benzbromarone, and lesinurad, enhances UA secretion. Clinical use of probenecid is typically contraindicated with creatinine clearance under 50 mL/min owing to diminished effectiveness. Advanced kidney dysfunction also limits the utility of probenecid and lesinurad, as their mechanism requires adequate renal function. Despite its potential effectiveness, benzbromarone usage is limited by safety considerations [60]. Probenecid, a uricosuric agent, enhances the excretion of UA through the kidneys. It is an alternative for patients unable to use XOIs. The comprehensive use of probenecid is not without consideration of adverse reactions, as it spans various organ systems [31]. Probenecid is effective in patients with normal renal function but carries the risk of causing kidney stones and gastrointestinal issues. Therefore, it is generally not recommended for patients with a history of recurrent kidney stones or renal impairment [61].

Long-term use of urate-lowering therapy, such as allopurinol or febuxostat, is essential to prevent recurrent gout attacks. However, long-term treatment often challenges patient adherence, especially as these medications must be taken daily, even without symptoms. Factors such as side effects, the complexity of the treatment regimen, and the prolonged duration of therapy can reduce patient motivation to continue treatment, increasing the risk of recurrent attacks if therapy is discontinued.

#### 2.2.3. Lifestyle Modifications

The prevalence rate of HUA has been significantly elevated worldwide due to lifestyle changes [53]. Preventive strategies for gout focus on lifestyle modifications and pharmacological treatment. Several medical conditions and lifestyle factors increase the risk of developing hyperuricemia, which can lead to gout.

Dietary intake significantly influences hyperuricemia; consuming foods high in purines elevates UA levels. Notable sources include organ meats (such as liver and kidney), sweetbreads, certain seafood (anchovies, sardines, mussels, scallops), and products like meat broths, gravies, dried beans, and peas [1,9,30,31,62]. Controlling the consumption of high-purine foods remains crucial for those susceptible to elevated UA levels and gout attacks. While dietary patterns emphasizing fruits, vegetables, and whole grains with minimal purines show therapeutic value, research reveals that, despite 75% medication compliance, patients did not experience fewer gout flares or decreased serum UA concentrations. Recurring gout episodes correlated with elevated SUA levels and sudden increases in dietary purine intake. This suggests that even compliant patients may require more aggressive urate-lowering therapy alongside strict dietary management to minimize recurrent attacks [62].

Similarly, consuming beverages with high fructose corn syrup, such as non-diet sodas, raises rapid UA production [12]. Beer’s elevated guanosine content and ethanol’s ATP degradation properties contribute to UA formation. Both alcoholic beverages and purine-rich foods (including animal proteins and marine products) increase gout susceptibility, historically linking it to affluent lifestyles. The molecular structure of sucrose combines glucose and fructose units [31]. Various alcoholic products, particularly beer, distilled spirits, and fructose-containing beverages, elevate UA concentrations. Minimizing consumption of these substances aids in UA control [63].

Obesity is a significant risk factor, as excess body weight can elevate UA levels. This investigation analyzed data from 29,310 individuals exceeding 20 years, with males comprising 14,268 participants. Statistical analysis, adjusted for relevant variables, indicated that heightened anthropometric measurements (body mass index (BMI), weight-adjusted waist index (WWI), and body roundness index (BRI)) exhibited strong positive associations with urate levels in a stepwise manner [64]. Conventional Mendelian Randomization (MR) analysis found a robust causal link between increased BMI, high-density lipoprotein (HDL), and systolic blood pressure (SBP) with gout risk. Fasting insulin, BMI, HDL, triglycerides (TGs), SBP, alanine aminotransferase (ALT), and serum urate were also causally related. Bidirectional causal effects were observed between HDL, serum urate, and gout. Results were consistent across the weighted median method and Mendelian Randomization Pleiotropy RESidual Sum and Outlier (MR–PRESSO) after outlier removal [65]

Certain medications, especially diuretics, can also influence urate levels in the blood, further increasing the risk of gout [66]. Pathological states involving rapid cellular destruction, specifically cancers of blood cells and widespread psoriasis potentially raise serum urate measurements and heighten gout risk among vulnerable populations [67].

Natural substances represent significant sources of physiologically active compounds. Evidence suggests that dietary intake of these bioactive elements offers defensive mechanisms against multiple conditions, particularly elevated UA levels. Plant-based polyphenols, specifically chlorogenic acid-related molecules, appear abundantly across everyday nutritional sources, including produce, beverages (tea and coffee), and cereal products [14,68]. Tea-derived polyphenolic compounds include epigallocatechin gallate and catechin. Research indicates that epigallocatechin gallate suppresses multiple inflammatory processes, including neutrophil migration induced by MSU, NLRP3 protein expression, and inflammatory mediator release, thereby reducing inflammation [3]. Research suggests that polyphenolic substances help alleviate elevated UA conditions by suppressing XO enzymatic function [53].

Research indicates that elevated beer intake correlates with increased serum urate concentrations in both sexes, while sake consumption shows no significant impact on urate levels. These findings suggest that both the specific type of alcoholic beverage and its ethanol content play distinct roles in hyperuricemia development [69]. This cohort study found a link between higher consumption of specific alcoholic beverages and increased gout risk for both genders. Differences in alcohol preferences between men and women may explain the variations in how total alcohol consumption affects each sex [70].

Alcohol, especially beer and spirits, and sugary drinks containing fructose can raise UA levels. Reducing or avoiding these can help manage UA levels [63]. Adequate hydration is crucial for diluting blood UA concentrations and supporting its excretion through the kidneys. Consuming at least 8–10 glasses of water daily can help reduce the risk of gout attacks.

Modern lifestyle modifications contribute to rising hyperuricemia cases, triggering various complications, including gout development, arthritic conditions, kidney dysfunction, and cardiac complications. The pathogenesis involves significant inflammatory processes, where disrupted immune cell function—particularly involving monocytes, macrophages, and T lymphocytes—plays a fundamental role in mediating inflammation [35].

### 2.3. Comorbidities

Male populations demonstrate significantly higher gout occurrence rates, exceeding female rates by 3.1 to 10.1 times. Recent years show escalating patterns in disease frequency and new cases, with occurrence rates reaching 11–13% and new diagnoses approaching 0.4% among octogenarians. Age-associated kidney dysfunction, modified drug pharmacokinetics, and multiple concurrent health conditions substantially impact therapeutic safety and efficacy in gout treatment [9].

Predisposing factors for gout development encompass hereditary components, metabolic characteristics, and concurrent health conditions, particularly metabolic disorders and cardio-renal pathologies [3,71]. Many common medical conditions can limit the treatment options available for managing gout. Diseases like kidney problems, heart failure, and diabetes may mean that standard gout medications, like NSAIDs or colchicine, are contraindicated or carry excessive risk for certain patients. The presence of multiple concurrent health issues frequently complicates the effective management of gout, as the limitations imposed by these comorbidities restrict the therapies that can be safely used. Clinicians must navigate these complexities to find appropriate gout treatment approaches for patients with additional underlying conditions [60]. Hyperuricemia is a risk factor for developing various chronic conditions, such as type 2 diabetes, joint edema, dyslipidemia, central obesity, renal failure, metabolic syndrome, and high blood pressure.

Gout patients, especially those with poorly controlled disease, had an increased prevalence of cardiovascular and bone disorders compared to gout-free individuals. Recognizing and tracking gout in CKD may improve outcomes, but over 33% of gout cases lacked a documented diagnosis [72]. Gout correlates with elevated risks of ischemic heart disease, myocardial infarction, and cerebrovascular disease [73,74]. Furthermore, recent gout exacerbations transiently increase the incidence of cardiovascular events.

Weight gain during adult years has been consistently linked to increased gout risk. Compared to the general population, gout patients had a higher prevalence of obesity, hypertension, CKD, diabetes mellitus, and hyperlipidemia. Body weight is a significant factor influencing serum urate levels, and weight reduction may help lower the risk of gout development [64,75,76].

Other comorbidities associated with gout include hypothyroidism, anemia, psoriasis, chronic pulmonary disease, osteoarthritis, and depression. Psoriasis contributes to hyperuricemia due to increased epidermal cell turnover and UA production. Simultaneously, CKD reduces urate excretion, leading to elevated UA levels and a higher risk of incident gout. The scarcity of studies specifically investigating urate-lowering therapies in CKD has led to inconsistent expert recommendations. Initiating allopurinol at low doses and gradually increasing the dosage can mitigate risks. Some CKD patients may require higher allopurinol doses to achieve urate targets. The safety of febuxostat in advanced CKD remains controversial, and the optimal management of gout in dialysis patients, including when to continue urate-lowering therapy, is unclear. Gout is frequently suboptimally treated in CKD and end-stage renal disease, emphasizing the need for further research to guide treatment in this population. Enhancing gout management can substantially reduce the burden of these comorbid conditions [60].

Gout is associated with higher overall mortality rates, including all-cause mortality and deaths attributed to specific causes, such as cardiovascular disease, infectious diseases, and cancer. In particular, gout has a strong correlation with increased cardiovascular mortality and contributes to mortality linked to renal disease, digestive disorders, and dementia.

The relationship between gout and dementia, including Parkinson’s disease, is intricate and not thoroughly understood. Research has yielded diverse findings, with some studies suggesting that hyperuricemia and gout are associated with a reduced risk of dementia, particularly Alzheimer’s disease. Conversely, contradictory evidence indicates that hyperuricemia and gout may increase the risk of dementia. Likewise, the association between gout and Parkinson’s disease remains inconclusive, with studies demonstrating inconsistent results, including lower, no specific, or higher risk of Parkinson’s disease in gout patients.

The UA pathway involves purine nucleotide phosphorylase and xanthine oxidase enzymes. Most mammals have functional uricase, which converts UA to soluble allantoin, effectively lowering UA levels. In humans, non-functional uricase leads to hyperuricemia and UA crystal accumulation. While UA has antioxidant and immune-protective properties, hyperuricemia causes gout, joint crystal deposition, nephrolithiasis, and chronic nephropathy and is linked to hypertension, metabolic syndrome, and cardiovascular diseases [18].

## 3. Molecular Mechanisms in the Treatment of Gout

Gout involves four main pathological processes: hyperuricemia, MSU crystal deposition, the body’s immune response to crystal deposition resulting in acute gout attacks, and advanced-stage tophi erosion of bone. UA, a product of purine metabolism, is primarily excreted by the intestines and kidneys. When serum urate levels exceed the saturation threshold, UA precipitates in the blood as needle-like crystals. The pathogenic pathway of gout involves phagocytosis of these crystals by immune cells, triggering an inflammatory response [20]

### 3.1. Hyperuricemia Treatment

#### 3.1.1. Inhibition of Uric Acid Production

UA is a weak acid with a pKa range of 5.75–10.3. At the physiological pH of 7.40, UA exists in its ionized form as MSU in blood and as potassium, ammonium, and calcium urate in urine. UA stone formation is favored in the urinary tract, where the pH can drop to 5.7. The usual range of UA is determined by the solubility limit of urate in body fluids. MSU has a solubility of approximately 7 mg/dL in connective tissue, which decreases at lower temperatures, such as those found in peripheral joints [18].

The enzymatic pathway of purine metabolism involves converting purines into hypoxanthine, which is oxidized by xanthine oxidase (XO) into xanthine and then further oxidized into UA. Elevated UA levels, if not adequately excreted, can lead to MSU crystallization [31,77,78].

XOIs, like allopurinol and febuxostat, are key drugs in managing hyperuricemia and preventing gout. Allopurinol, a purine analog, inhibits xanthine oxidase, reducing UA production by blocking the conversion of hypoxanthine and xanthine [16,39,60]. Recent drug developments focus on optimizing formulations and dosing regimens to improve efficacy and reduce side effects. Genetic factors influencing individual responses to these drugs are also under investigation to personalize treatment strategies.

#### 3.1.2. Uricosuric Agents

Uricosuric agents are medications that facilitate the elimination of excess UA by enhancing its excretion through urine, thereby maintaining serum UA levels within a normal range [6]. Some common ones include Probenecid, which blocks the reabsorption of UA in the kidneys, and Lesinurad, a newer drug that targets specific transporters, like URAT1, to boost UA clearance. Benzbromarone is another option that works similarly but is super potent compared to the others [1,15,38].

These medications are particularly effective for individuals with impaired UA excretion, commonly referred to as “under-excretion”. They are often combined with xanthine oxidase inhibitors (XOIs), such as allopurinol, to enhance therapeutic efficacy. However, careful monitoring is required due to potential side effects, such as an increased risk of kidney stone formation. The primary advantage of uricosuric agents lies in their effectiveness in patients with impaired UA excretion, making them a viable option for reducing serum UA levels. They also complement XOIs in combination therapy for better management of gout. Additionally, non-purine-based treatments provide an alternative approach to controlling UA levels without the purine-related side effects seen in some other medications [79].

A significant drawback of these agents is the increased risk of kidney stone formation, which results from elevated UA excretion in the urine. They are less effective in patients with CKD or those who overproduce UA, limiting their applicability [80,81]. Furthermore, side effects, such as gastrointestinal issues or rare but severe reactions, may occur with specific agents like sulfinpyrazone, making them less commonly used.

XOIs remain the safest and most effective SUA-lowering drug for chronic treatment, while the efficacy of uricosuric agents is strongly modulated by pharmacogenetics [15].

### 3.2. Modulation of Inflammation

GA is an inflammatory disease caused by UA metabolism disorder, leading to urate deposition and gout flares [2,36,60,72]. The innate immune system’s activation is central to GA pathogenesis, initiated through pattern recognition receptors (PRRs) such as NLRs, RLRs, and TLRs. The NLRP3 inflammasome, a multi-protein platform, plays a crucial role in this process. Its activation is triggered by K^+^ efflux, Ca^2+^ mobilization, lysosomal destabilization, and reactive oxygen species (ROS) generation. Thus, it leads to the processing and release of pro-inflammatory cytokines, including IL-1β and IL-18, and the activation of inflammatory transcription factors, such as NF-κB and MAPKs [2,31]. NLPR3 is an intracellular molecular platform that responds to multiple danger signals and is implicated in inflammasome activation. ROS effects vary based on concentration: at high concentrations, they disrupt cellular organelles; at low concentrations, they are signal molecules in immunity [32].

MSU crystals act as damage-associated molecular patterns (DAMPs) in gout, triggering NLRP3 inflammasome activation in mononuclear phagocytes. This initiates an acute gout attack, releasing inflammatory cytokines, especially IL-1β and IL-18. ROS participate in various pathways, i.e., Nrf2-Keap1 and ROS-autophagy reduce oxidative stress and inflammation, while ROS-NF-κB-NLRP3, ROS-MAPK, ROS-NET, and ROS-Ferroptosis activate inflammation and apoptosis. The IL-1β pathway has become a key therapeutic target, with IL-1 inhibitors like anakinra and canakinumab showing efficacy in treating acute and chronic GA [82,83].

MicroRNAs (miRNAs), small non-coding RNAs that regulate protein expression, may influence GA onset and progression by modulating immune function and inflammatory responses [30]. While progress has been made in understanding gout pathogenesis, questions remain about cellular and molecular mechanisms. Future research will provide insights into complex intracellular signal components after MSU crystal uptake, such as mitochondrial oxidative stress, thioredoxin-interacting protein TXNIP, and (Never in Mitosis Gene A(NIMA)- related kinase 7) NEK7. Understanding the interplay between ROS, miRNAs, the NLRP3 inflammasome, and various inflammatory pathways will be crucial in developing more targeted and effective treatments for GA.

Neutrophil extracellular traps (NETs) play a crucial role in GA, but researching them presents significant challenges. Neutrophils, the primary cells involved in NET formation, have a short lifespan of up to 5 days in vitro and are notably fragile. While HL-60 cells are sometimes used as alternatives, they have limitations in NET generation and differ from neutrophils in various cellular functions. NETs, complex structures composed of DNA, histones, and enzymes, like myeloperoxidase and elastases, exhibit a dual role in GA. They can trigger inflammation by releasing cytokines but, when aggregated, they may also help resolve inflammation by sequestering and degrading MSU crystals. Current treatments for GA include NSAIDs (which inhibit COX enzymes), colchicine (disrupting neutrophil function), corticosteroids (suppressing immune response), and anakinra (blocking IL-1β). More research is planned on developing neutrophil survival conditions in vitro, enhancing HL-60 cell models, providing further insight into the NET-targeted approach in healing, and how aggregated NET protects tissues. Such work could result in better drug therapies for GA and improved insight into NET-related inflammatory mechanisms [6].

### 3.3. Erosion of Bone (Stage of Tophi)

Gout is an inflammatory disease due to the deposition of MSU crystals in cartilage, joints and soft tissue, and is a complication of chronic hyperuricemia [3]. Tophus, characteristic of chronic gout, is a dense, soft tissue lesion seen as a granulomatous response to MSU crystals. Tophaceous deposits may lyse into the bone, cartilage, and tendons, which have a definite structural impact [5,6,7]. The mechanism, therefore, through which bone erosions develop in chronic gout is still poorly understood. MSU crystal deposition seems to promote the development of bone erosions to a different extent depending on the joint involvement of MSU crystals. The intraosseous tophi contribute most to bone erosions, followed by intra-articular and periarticular tophi. This study reveals that MSU crystal deposition promotes the development of bone erosions to varying degrees, depending on the anatomic location of the deposited crystals. Intraosseous tophi have the most significant impact on bone erosion, followed by intra-articular and periarticular tophi [84]. Prolonged deposition of amounts of urate crystals in a joint cavity leads to bone erosion, which progressively causes skeletal muscle necrosis and joint deformity [44]. The bone erosion group, compared to the non-bone erosion group, exhibited older age, longer duration of gout and tophi, higher serum creatinine levels, a higher proportion of individuals with a history of drinking and ulceration, and a lower glomerular filtration rate (GFR). Univariate logistic regression analysis revealed that sex, age, body mass index (BMI), gout duration, tophi duration, glomerular filtration rate (GFR), white blood cell (WBC) count, serum creatinine (sCr) level, smoking history, drinking history, and presence of ulceration were associated with bone destruction. Multivariable logistic regression analysis further demonstrated that tophi duration, drinking history, ulceration, and sCr were positively and independently related to bone erosion [44].

Without proper treatment, acute gout attacks become more frequent, and urate crystal deposition can result in tophi formation and bone erosion, leading to joint damage, deformity, and disability. Tophi are inflammatory lesions that form near MSU crystal deposits and consist of chronic inflammatory tissue with MSU crystal accumulation. Joint cartilage destruction is a common issue in tophaceous gout. Tophi, characterized by a soft tissue component, are closely associated with MSU crystals and contribute to bone destruction in gout. Therefore, it is crucial to investigate additional risk factors related to bone destruction in gout patients.

All of them have indicated that early identification and intervention of tophi is crucial in preventing the development of bone erosions. Urate and soft tissue components of the tophus are strongly and independently associated with bone erosion in gout [85].

Tophaceous gout frequently leads to bone damage. A tophus is a well-structured formation with a core of MSU crystals surrounded by soft tissue containing innate and adaptive immune cells, especially macrophages. Bone damage in tophaceous gout is caused by an imbalance in bone remodeling at the bone–tophus interface. Excessive bone resorption by osteoclasts, not sufficiently counterbalanced by osteoblast-mediated bone formation, results in bone erosion at the tophus–bone interface. Conversely, abnormal osteoblast activity may stimulate new bone formation, causing sclerosis and spur formation [45].

Conditioned medium from MSU crystal-stimulated RAW264.7 macrophages suppresses the expression and secretion of osteoprotegerin (OPG) while inducing the expression and secretion of proinflammatory mediators in MC3T3-E1 cells [45]. Conditioned medium from MSU crystal-stimulated Tsuchiya Human Peripheral blood-derived-1 (THP-1) monocytes slightly affects the expression of osteoblast marker genes but increases the expression of proinflammatory mediators in human primary osteoblasts. Factors secreted by MSU crystal-stimulated macrophages reduce osteoblast differentiation and induce the expression and secretion of proinflammatory mediators from osteoblasts. These findings suggest that the disordered bone remodeling and bone erosion in joints affected by tophaceous gout result from both direct and indirect effects of MSU crystals [45]. There is a strong relationship between bone erosion and the presence of intraosseous tophus. Tophus infiltration into bone is the primary mechanism for bone erosion and joint damage in gout. In rheumatoid arthritis, periarticular cortical bone erosion results from excessive bone resorption and insufficient bone formation, triggered by synovitis, proinflammatory cytokines, RANKL, and anti-citrullinated protein antibodies. These factors promote osteoclast differentiation and local bone resorption. Despite current therapy inhibiting erosion and inflammation, repair of existing lesions rarely occurs, partly due to proinflammatory cytokines suppressing bone formation [86].

### 3.4. Genetic Variations

The patient’s severe hyperuricemia and gout are likely to be due to a combination of genetic mutations and obesity. Mutations in ABCG2 and SLC16A9/MCT9 were detected, with ABCG2 dysfunction significantly reducing UA transport and increasing gout risk. Matsuo et al. reported that ABCG2 mutations lead to a 75% loss of function, raising gout risk (OR 25.8), and severe dysfunction also results in early-onset gout [43,87].

The patient’s ABCG2 and SLC16A9/MCT9 mutations, combined with severe obesity, visceral fat, hyperinsulinemia, and low adiponectin, are likely to have contributed to his severe hyperuricemia and early gouty tophi with bone lesions, causing gait disturbance within six years. Early diagnosis and timely treatment of hyperuricemia are essential, especially in severely obese patients with arthritis [43].

Variations in genes related to UA metabolism, such as those encoding xanthine oxidase or UA transporters, can influence individual responses to gout treatments. Research is ongoing to identify genetic markers associated with gout severity and treatment. Epigenetic changes, including DNA methylation and histone modifications, can affect gene expression related to inflammation and UA metabolism. These modifications may influence susceptibility to gout and responses to treatment [88,89].

## 4. Nanoparticles in Gout Treatment

Nanotechnology involves handling particles at the atomic–molecular level, producing 1–100 mm nanoparticles. They can be used for many purposes due to the large surface areas and characteristic improvements of these particles in many industries, such as pharmaceutical and electronics. Due to the ability to work with the materials at the nanometre scale, various fields have opened new frontiers, such as energy, nature and life sciences, agriculture, construction, transportation, and environmental protection, also providing great potential to enhance human quality of life [90,91].

Thus, nanotechnology promises much in many application areas but should be used cautiously because of its effects on health and the environment. To reverse this trend, much research needs to be done, and correct regulation put in place to harness the benefits and prevent the harms that may come along with the use of social networks. Nanocarriers reflect new approaches in gout treatment, increase drug solubility, exhibit anti-inflammatory properties, change the properties of UA crystals, and assist in diagnostics, as shown in Table 1.

The study discovered that, although the nanoparticles were not superior to allopurinol in terms of UA lowering for patients with GA, they had fewer side effects in terms of damage to the kidney and liver and the relative levels of lipids. It is essential to note that these nanoparticles were able to minimize joint swelling and the diameter of the ankle. Zinc oxide nanoparticles are specifically used to reduce oxidative stress and treat GA. This infers that particle drugs may produce favorable directions for patients with fundamental responses to conventional compounds and may aid in the chronic control of the ailment [57]. Nanotechnology holds much promise for one particular therapy—GA. Said particles have demonstrated the ability to lower UA in rodents, thus ushering in the likelihood of further creation of safer, more efficient drugs. For instance, turmeric nanoparticles (T-NPs) have shown possibilities for designing effective anti-gout agents. Given this, these T-NPs can cross intestinal barriers and manage the high UA levels central to gout control. By comparing the reduction effect of UA concentrations after treatment with T-NPs to that of marketable drugs, it became clear that T-NPs have the best prospects for managing gout. T-NPs demonstrate potential as safe, effective anti-gout therapeutics [7,20].

The anti-inflammatory effectiveness of AuNPs against these diseases depends on properties such as size and shape, biocompatibility, and drug-delivery ability due to functionalization by biological macromolecules and ligands [98]. Several issues have been identified in the science of metal NP synthesis and application that require solution, including the difficulty in processing the particles to the appropriate size and shape. A common characteristic of many methods for making metal NPs is high temperatures and stringent chemical conditions, which are difficult to apply in large-scale production. Altogether, the size and shape of the metal NPs play a crucial role in determining their characteristics and possible uses, so NP synthesis should be designed concerning size and shape control [99].

Studies evaluating joint swelling across 13 trials demonstrated five nanoparticle types (*Aurantii fructus immaturus carbonisata*-derived carbon dots [AFIC-CDs], copper oxide nanoparticles [CuO-NPs], nano Ginsenoside Rb1 [nano-GsRb1], PLR-CDs, and interleukin-1Ra bio-nanoparticles [IK-NPs]) effectively reduced ankle inflammation. Biochemical analyses showed decreased UA, creatinine, lipid profiles, and bilirubin levels. Nano-formulations exhibited superior safety compared to allopurinol, maintaining normal organ function. Heterogeneity factors included particle composition, dosage, and animal characteristics [7].

Nanomaterials have paved the way for more effective and targeted therapies in medicine. For example, nanomaterials protect miRNAs in cancer and inflammatory disease therapies, allowing direct drug delivery to target cells. However, using nanomaterials also poses challenges, such as potential side effects in immune responses. Further research is needed to ensure the safety and effectiveness of nanomaterial-based therapies. Meanwhile, gene editing techniques, such as CRISPR/Cas9, also offer the potential to treat diseases through miRNA downregulation [7,30]. Targeted delivery requires selective drug accumulation at intended sites, maintaining optimal circulation time while evading immune response, with specific tissue targeting and controlled release properties [61].

Treating UA nephrolithiasis with allopurinol requires high drug levels in the kidneys, which can result in harmful side effects elsewhere in the body. Conventional treatments lack precision in targeting specific organs without causing toxicity. Nanotechnology emerges as a promising strategy, enabling precise drug delivery to organs like the kidneys and liver, maximizing therapeutic effects while reducing systemic side effects. Kandav et al. show that the specific accumulation of ABNPsopt in the kidney could be attributed to the specificity of polymer for cubilin and megalin receptors [94].

Nanosystems show promising kidney-targeting capabilities: PEGylated gold nanoparticles (75 ± 25 nm) and mesoscale particles (400 nm) demonstrated specific kidney accumulation, with the latter showing 7-fold higher kidney uptake. Low MW chitosan is particularly effective, with studies showing 13-fold increased kidney accumulation when conjugated with prednisolone due to specific uptake by megalin receptors in renal tubular cells [95].

Nanoparticles advance gout therapy through multiple mechanisms, including enhanced drug delivery, inflammation modulation, UA crystal modification, and improved diagnostics. The main advantages of nanoparticles lie in their ability to improve drug pharmacokinetics, reduce toxicity, and enhance therapeutic efficacy. However, regulatory and safety aspects and the need for further research, including comprehensive clinical trials, must be addressed. By continuing to develop and refine nanoparticle-based therapies, we can look forward to a more personalized, effective, and safe future for gout treatment.

Nanoparticle advantages comprise the following:

(a) Nanoparticle efficacy depends on encapsulated drug properties and carrier material characteristics. These substances were analgesic, had anti-inflammation properties, and controlled oxidative stress. The nanomaterial shell prolonged the biological half-life and improved the pharmacokinetics of encapsulated substances to maximize their therapeutic effect. However, traditional drug treatment strategies show low efficacy and safety due to the (bio)pharmaceutical shortcomings of these drugs, such as poor chemical stability and limited ability to target the pathophysiological pathways [23].
(b) Nanocarriers enhance gout treatment by improving drug bioavailability, particularly for non-metallic compounds. These systems provide superior solubility, reduced drug loss, and targeted delivery, optimizing therapeutic efficacy while minimizing side effects.
(c) Nanoparticles offer a promising approach to targeted drug delivery for gout treatment, surpassing traditional medications, like allopurinol, in safety and efficacy. Research indicates that treatments that use nanoparticles affect the kidney and liver less than most other treatments. One example showed how copper sulfate that has been entrapped in nanoparticles could lower UA levels in test animals by about 90%, with less toxicity to the organs than free copper sulfate. In addition to achieving improved targeting and protective encapsulation that ensures high therapeutic outcomes, these nanoparticle-based systems reduce the therapy’s side effects, thereby exhibiting promising strategies for new-era gout treatment, and probably for other chronic diseases. Supercontrol of the biodistribution of drugs results in numerous side effects, which makes the control of gout through drugs a massive challenge. These nanocarrier systems are also employed to overcome the restrictions of today’s drugs, not only the problem of biodistribution but also that of the stability and solubility of the drugs [21].
(d) The application of nanoparticles in gout treatment increases its effectiveness and minimizes the side effects of the drug through better delivery mechanisms. They allow for the extensive control option because they target inflammation and UA production at the same time. Nano could load several therapeutic agents to achieve fast relief from inflammation and decrease UA levels from a single delivering system. This approach promises to provide better gout treatments and diagnoses, thus revolutionizing treatment processes.

Nanoparticles show promise for effective GA treatment due to their safety and efficacy. However, it is necessary to conduct more research on this topic. There is an urgent need for larger sample sizes, more extended trial periods, and well-designed preclinical and clinical trials to establish their efficacy conclusively. The researchers also need to pay attention to the issues related to identifying the proper dosage of the drugs based on nanoparticles. The high heterogeneity in animal quantities across studies has raised concerns about experimental applicability, highlighting the importance of establishing appropriate animal numbers for nanomedicine experiments. These efforts will help solidify the potential of nanoparticles in gout treatment and ensure their successful translation to clinical practice.

Preclinical studies show that therapies utilizing nanocarriers effectively minimize inflammation through selective delivery to affected tissues, blocking UA crystallization and enhancing joint performance in gout-induced animal models [21].

## 5. Future Developments in Gout Treatment

Recent advances emphasize a comprehensive approach to managing gout that combines curative treatments, preventive measures, and lifestyle modifications. This integrated strategy involves patient engagement in treatment planning and adherence to lifestyle changes for optimal outcomes. Gout presents clinically as self-limiting acute arthritis with intense pain. Without proper treatment, it can progress to joint mobility restrictions and severe tissue deterioration, significantly compromising patients’ quality of life [23].

Research demonstrates a higher prevalence of GA among women compared to men, potentially linked to reduced estrogen levels, particularly affecting postmenopausal women. Experimental findings support this observation, showing enhanced treatment responses in mixed-gender animal groups versus male-only subjects.

Effective management of gout involves a comprehensive approach, including comorbidity screening, management of acute attacks, urate-lowering therapy, and anti-inflammatory preventive measures [21]. Therefore, well-designed long-term clinical trials are needed to evaluate the efficacy and tolerability of various gout treatments [29]. When MSU crystals remain undetected, gout diagnosis typically depends on analyzing clinical indicators, laboratory data, and imaging outcomes. The key factor triggering arthritic manifestations is hyperuricemia, for which detailed treatment protocols exist [4].

Experimental studies of GA utilize various animal models to explore the inflammatory mechanisms responsible for skeletal and cartilage deterioration, representing a crucial step in therapeutic development. The research predominantly employs four distinct approaches: hyperuricemia induction through potassium oxonate administration, GA development via MSU crystal introduction, acute GA with elevated UA levels, and hyperuricemia establishment through dietary modification [9]. Several animal species demonstrate utility in this research, with chickens emerging as an optimal model when MSU is administered into their left ankle joint for studying GA. Additional experimental models include mice, rabbits, and zebrafish, each contributing to gout research investigations. This animal experimentation phase remains essential for understanding disease mechanisms and validating potential treatments before human clinical trials.

Research is exploring anti-hyperuricemia compounds extracted from natural plant sources as therapeutic alternatives, given their reduced adverse effects compared to conventional treatments [100,101]. Current research examines medicinal plants’ anti-inflammatory properties in arthritis treatment, focusing on improving their bioavailability and reducing side effects. These herbal compounds’ effectiveness can be enhanced by understanding their mechanisms and implementing advanced delivery systems, like liposomes, nanoparticles, and transdermal methods [20]. Nanoparticles, such as ZnO-NPs, can potentially reduce UA and cholesterol levels, but their side effects need further investigation [90]. Herbal therapies, which for years now have been a natural treatment preferred to synthetic drugs, have poor absorptive active ingredients [20]. While Traditional Chinese Medicine (TCM) offers comprehensive treatment approaches, it faces limitations, including challenges in personalizing therapies and requiring practitioners to possess extensive specialized knowledge [1]. New gout treatments require long-term trials to assess therapeutic effectiveness. Despite development challenges, herbal medicines and TCM warrant investigation. Both natural compounds and ncRNAs offer untapped potential for GA drug development, mainly through NLRP3 regulation, though further research on their efficacy and clinical applications remains necessary.

Gene therapy, especially therapies targeting NLRP3, also holds a lot of promise but has some limitations in its structure. Delivery systems’ stability, safety, and efficiency need to be further addressed. In conclusion, future research and development advancement is believed to improve GA treatment through different models depending on natural products, ncRNAs, new chemicals, and gene therapy [5]. Emerging treatments include biologics targeting specific inflammatory pathways in gout and gene therapies aiming to modify the body’s response to MSU crystals.

The future of gout management lies in personalized treatment approaches, as demonstrated by successful trials of canakinumab, an IL-1β antibody targeting inflammatory molecules. While CRISPR/Cas9 research explores genetic manipulation of UA metabolism for potential cures, treatment customization based on individual genetic profiles and biomarkers aims to minimize side effects. Identified genetic variations affecting UA metabolism enable more precise interventions. Innovative technologies and nano-delivery systems, including liposomal and dendrimer particles, enhance drug effectiveness in affected joints. Emerging alternatives, like stem cell therapy for cartilage repair, targeted dietary interventions, and advanced monitoring technologies through wearable devices, offer promising treatment options and improved disease management. The IL-1 inhibitors are a recent addition to the armamentarium of drugs used in therapy for acute attacks of gout for refractory patients and patients with comorbidities, where using NSAIDs, Colchicine, and glucocorticoids is not possible [18].

Studies revealed that nanoparticle therapy effectively lowered cholesterol and LDL concentrations compared to control groups. Nevertheless, both nanoparticle and allopurinol treatments resulted in elevated liver enzyme levels (ALT, AST, and ALP) [90].

Evidence shows that progress in the diagnostic and therapeutic approach to gout has been achieved. DECT and ultrasound yield better and less invasive methods for the diagnosis of gout and monitor disease activity than conventional imaging [55].

Novel pharmacologic approaches present improved target therapies and various new agents as urate-lowering therapies augment management and treatment choices for gout, especially for patients with nonresponsive or chronic gout conditions. Moreover, trends in pharmacogenomics and molecular treatment are helping to define individual patient reactions to medicines and to suggest the development of selective therapeutic strategies.

Nanotechnology emerges as an innovative approach to gout treatment, with nanoparticles offering enhanced drug delivery due to their small size and extensive surface area. Nanomedicine has enhanced the pharmacological properties of conventional anti-inflammatory arthritis treatments. Their advantages include targeted delivery of anti-inflammatory agents to affected joints, inhibition of urate crystal formation, and enhanced drug solubility. Notably, nanoparticles improve herbal medicine efficacy by enhancing bioavailability and ensuring precise delivery to target sites. They also serve as effective carriers for gene therapy delivery. These technological advances suggest that future gout therapies, particularly those incorporating herbal medicines, will be more efficient and personalized through improved targeted delivery systems. Nanoparticles are safe medications for GA, which can effectively reduce UA levels in rodents [7]. Studies show that C. colocynthis extract and CC-AgNPs effectively inhibit protein denaturation and stabilize RBC membranes, while phyllum tomentosum-derived silver nanoparticles demonstrate anti-inflammatory effects through multiple mechanisms. Research indicates that plant-based AgNPs exhibit superior antiarthritic properties due to their nano-scale dimensions and biological interactions. Smaller nanoparticles (<400 nm) show enhanced cellular uptake by inflammatory cells and better penetration into arthritic tissue. Additionally, a novel mRNA-based treatment approach (mUox@iLAND) addresses the evolutionary loss of urate oxidase in humans and apes, utilizing lipid nanoparticles to decrease serum UA levels through hepatic protein expression in murine models [9].

Artificial Intelligence and Machine Learning technologies are revolutionizing gout management by analyzing extensive data from genetic studies, biomarker research, and clinical trials, with recent ML algorithms successfully predicting gout attack risks based on clinical and lifestyle factors. Future treatments will likely combine multiple approaches, including biologics, gene therapy, and enhanced traditional medicines, as research shows greater effectiveness of combination therapies in certain patients. Digital health technologies are improving preventive care and patient education through mobile applications that monitor lifestyle changes, dietary habits, and symptoms, leading to better treatment adherence. The rapid advancement across multiple fields suggests promising developments in gout management, as the collaboration between scientists, clinicians, and technology aims to improve patients’ quality of life.

## 6. Conclusions

Effective gout management requires a multifaceted approach combining acute attack treatment and long-term prevention strategies. Integrating lifestyle modifications alongside pharmacological interventions is crucial for achieving optimal disease control and improving patient outcomes. The future of gout treatment is bright, with promising advancements in nanoparticle technology, biological therapies, and diagnostic tools. Continuous research and development in these areas will undoubtedly lead to more effective and personalized gout care, ultimately enhancing the quality of life for individuals with this condition. Embracing innovation and evidence-based practice will drive progress and achieve better outcomes for gout patients.

## Figures and Tables

**Table 1:** Nanoparticle Delivery Systems in Relation to GA.

No	Drug	Materials/Route of Administration	Characteristic	Physicochemical Enhancement	Pharmacological Enhancement	Ref
1	Shogaol	SLN preparation utilizes pressure homogenization techniques, combining triglyceride-monostearate lipid matrix with a dual emulsifier system (span/tween 80). SLN was administered intragastrically in Male Sprague Dawley (SD) rats.	The SLNs produced were small (<100 nm), spherical, and smooth, with negative zeta potential (−15.2 mV), high encapsulation efficiency (87.67%), and acceptable polydispersity.	The SLNs demonstrated improved in vitro release profiles and enhanced oral bioavailability compared to the free drug.	In hyperuricemic models, SLNs exhibited enhanced UA-lowering effects through enzyme inhibition and cytokine reduction, surpassing free drug performance.	[92]
2	Colchicine	Chitosan nanocarriers containing colchicine were synthesized via the spontaneous emulsion method. NP gel was administered topically in albino rabbits.	Optimized CHNPs demonstrated 294 ± 3.75 nm diameter, with 92.89 ± 1.1% entrapment and 83.45 ± 2.5% drug loading.	In vitro release: 89.34 ± 2.90% over 24 h.	Colchicine-loaded CHNPgel outperformed plain colchicine, showing potential as an efficient gout treatment delivery system.	[93]
3	Turmeric	Solvent evaporation or nano-precipitation technique. NPs were orally administered.	T-NPs: ~46 nm size, +29.55 ± 3.44 zeta potential, 0.264 PDI.	T-NPs are designed for enhanced oral solubility.	T-NPs significantly reduced UA levels, showing superior potential for gout management.	[20]
4	Zinc oxide	Precipitation method. NPs were orally administered in Balb/C mice.	SEM analysis showed zinc oxide nanoparticles: ~37 nm, amorphous morphology.	ZnO-NPs significantly reduced urea, creatinine, and UA in gout-induced mice (*p* < 0.001).	ZnO nanoparticles (10, 20 ppm) significantly reduced serum UA (*p* < 0.001), in treating GA. They decreased ROS and Thiobarbituric Acid Reactive Substances (TBARS) and improved blood count and Liver Function Tests (LFTs), indicating reduced hyperuricemia. Histopathology showed no changes in liver, kidney, or muscle tissues.	[90]
5	Allopurinol	Allopurinol-BSA nanoparticles (ABNPsopt) prepared via desolvation. NPs were intravenously administered in Swiss albino mice.	The 13 ABNP batches showed particle sizes ranging from 220.1 to 358.6 nm, with PDI values between 0.155 and 0.499. The zeta potential varied from −34.1 to −21.7 mV, while encapsulation efficiency ranged from 10.2% to 67.5%.	ABNPsopt demonstrated superior kidney targeting, achieving 21.26-fold higher drug levels than serum, whereas free drug showed no tissue retention after two h.	ABNPsopt enhanced renal allopurinol uptake through cubilin/megalin receptor recognition of albumin carrier, offering improved therapeutic strategy for hyperuricemic nephrolithiasis.	[33]
6	Allopurinol	Allopurinol-loaded chitosan nanoparticles (A-CNPs). NPs were intra-venously administered in Swiss albino mice.	A-CNPs showed a size of 375.3 ± 10.1 nm, PDI of 0.362 ± 0.01, and ZP of 32.5 ± 2.7 mV. The low PDI and High positive ZP indicates a stable, monodisperse formulation.	Drug release from A-CNPs fits the Higuchi model (R^2^ = 0.9916) with a release exponent (n) of 0.59. This indicates diffusion-controlled, non-Fickian release kinetics.	Low molecular weight chitosan demonstrated enhanced renal targeting through megalin receptor-mediated uptake and increased solubility, optimizing drug delivery for hyperuricemic nephrolithiasis treatment.	[94,95]
7	Allopurinol-loaded chitosan-coated magnetic nanoparticles (A-MNPs)	Magnetic iron oxide nanoparticles synthesized through co-precipitation technique. NPs were intra-venous administered in Swiss albino mice.	All magnetic nanoparticles were below 250 nm with low PDI, indicating homogeneous dispersions. High zeta potential values suggested good stability. Size increases in C-MNPs and A-MNPs confirmed successful polymer coating and drug loading. A-MNPs showed 57.55 ± 0.05% entrapment and 27.35 ± 0.02% loading efficiency.	Drug release from A-MNPs best fits the Higuchi model, with a release exponent (n) of 0.67, indicating diffusion-controlled, non-Fickian release through the swollen polymer matrix. This pattern matches previous findings with chitosan nanoparticles.	A-MNPs were developed using chitosan coating to protect from early clearance and enable kidney targeting. The formulation achieved 19.07-fold higher drug concentration in kidneys than serum, with sustained release demonstrated through in vivo and histological studies for treating hyperuricemic nephrolithiasis.	[96]
8	Ethanolic fruit extract of Citrullus colocynthis (C. colocynthis) (EFECC) and synthesized silver nanoparticles (CC-AgNPs)	C. colocynthis fruit extract mediated AgNP synthesis. NPs activity tested in vitro.	Microscopy revealed spherical CC-AgNPs (10–45 nm), crystallinity confirmed via XRD analysis. FTIR indicated phenolic compounds and metabolite functionalization.	Synthesized nanoparticles exhibited enhanced antioxidant activity versus crude extract while demonstrating multi-target anti-gout effects through membrane protection, enzyme inhibition, and protein stabilization.	CC-AgNPs exhibited superior antioxidant capacity and anti-arthritic properties versus fruit extract, demonstrating enhanced inhibition of xanthine oxidase, protein denaturation, and membrane damage.	[68]
9	Urate oxidase mRNA encapsulated in ionizable lipid nanocarriers (mUox@iLAND).	Ionizable lipid carriers loaded with dual nucleic acids: luciferase mRNA and fluorescent siRNA. Intravenously administered to HU mice.	Nanocarrier analysis revealed size ~169 nm, uniformity index 0.148, surface charge −1.22 mV, and mRNA loading of 94.2%.	iLAND internalization occurs via multiple routes: caveolae, clathrin-dependent endocytosis, macropinocytosis, and independent mechanisms.	The Nanocarrier system employs receptor-mediated internalization and endosomal release, providing sustained therapeutic effects. Unlike allopurinol’s acute response, mUox@iLAND achieved prolonged UA reduction through controlled delivery.	[97]
